# Medical Items

**Published:** 1916-08

**Authors:** 


### MEDICAL ITEMS

An illuminating pamphlet on Pollen Extracts and their adaptability to the prophylaxis and treatment of hay fever comes from the press of Parke, Davis & Co.

"As regards the symptom complex known as hay fever," says the booklet by way of introduction, "there is no doubt in the minds of the majority of authorities at the present time that it emanates from the pollens of the flowers of various grasses, shrubs and trees. Elliotson, in the early part of this century was the first to suggest the relation of the pollens of grasses to hay-fever, but it was left for Blackley and later Dunbar and his pupils to definitely prove in a scientific manner this relationship.

"At present the pollen diseases are defined as a group of vasomotor disturbances, of seasonal periodicity, depending upon individual hypersensitiveness to the pollens of certain plants, and characterized by exudative catarrhal inflammation of the nasal, tracheo-bronchial, and conjunctival mucous membranes. In America two varieties of hay-fever are recognized—the spring variety, due to the Graminaceae, especially timothy grass, and the autumnal variety, due to the Compositae, especially the ragweeds. * * * *

"It has also been established by Freeman, Goodale and others, as a result of much experimental and clinical work, that individuals who are susceptible to the proteid of one pollen are sensitive to proteids of other pollens of the same family, and that protection can be produced in the majority of patients by immunization with the extracts of the pollen of the most frequently encountered representative members of that family. Hence, Ragweed Pollen Extract will protect against members of the family of Compositae, and Timothy Pollen Extract will protect against members of the family of Graminaceae. These two extracts, therefore, will be found suitable for prophylaxis and treatment for the large majority of cases of hay-fever encountered in America."

In addition to the two extracts mentioned in the foregoing, announcement is made of a third product, Pollen Extract Combined. The three varieties are briefly described as follows:

"1. Timothy Pollen Extract, for the estimation, prophylaxis and treatment of the spring or vernal variety of hay-fever.

"2. Ragweed Pollen Extract, for the estimation, prophylaxis and treatment of the autumnal variety of hay-fever.

"3. Pollen Extract Combined, which may be used in either vernal or autumnal hay fever, but is especially indicated in cases which begin early and last long, showing susceptibility to the early and late pollens."

The prophylactic and therapeutic use of the extracts is, of course, fully covered in the pamphlet, which also contains excerpts from articles by various well-known authorities—Ulrich of Minnesota, Freeman of London, Lowdermilk of Kansas, Koessler of Rush Medical College (Chicago), Cooke of New York City, and others. It is not extravagant to say that the booklet which bears the title "Pollen Extracts," is a valuable contribution to our current literature on the subject of hay fever. A copy of it may be obtained on request from Parke, Davis & Co., Detroit.

---

The increasing knowledge of endamebas and their relation to infections and other pathological conditions calls for an extension of ipecac therapy. The alkaloids of ipecac are specific for the endamebas and quickly destroy all motile forms but if given by mouth they cause great nausea. The hypodermatic injection of emetine is efficient but time-consuming, frequently painful and expensive.

Alcresta Tablets of Ipecac, Lilly, have made it possible to administer the ipecac alkaloids by mouth in massive doses without causing vomiting or nausea. These tablets are uncoated and disintegrating but do not liberate the alkaloids until the compound reaches the alkaline intestinal juices. Thus vomiting and nausea are avoided. Both physicians and dentists are using these tablets with much success in dysentery and pyorrhea and also in many affections that are known to have their course in local foci of infection.

---

### PITUITRIN IN UTERINE INERTIA.

Administered hypodermically during the second stage of parturition (it should not be given during the first stage), Pituitrin is said to convert a case of tedious inertia into one of normal rhythmic labor, saving time, preventing suffering on the part of the mother, and diminishing the risk to the child which attends upon protracted labor. Furthermore, in many cases it obviates the use of forceps.

Pituitrin is a pituitary extract, otherwise described as a solution of the active principle of the infundibular porton of the pituitary gland (in animals). This gland, as is well known, lies at the base of the brain.

There are a number of pituitary extracts on the markets—products, it may be said in all fairness, not equal in therapeutic efficacy. To be of definite value as an oxytoxic it is obvious that the solution or extract must be highly active. Owing to unavoidable variations in fresh glandular tissue, the amount of gland substance represented in the preparation is not an accurate index of its strength. Assurance of therapeutic activity can be obtained only by rigid assay. In view of these facts a recent statement of Parke, Davis & Co. with respect to their Pituitrin is peculiarly significant:

"Because of its importance in obstetrical practice we have given much attention to a determination of the proper strength and standardization of Pituitrin. The result of our investigations is a product of high potency, representing the average activity of 0.2 gramme of fresh posterior pituitary lobe to each c.c. of the solution. As an oxytoxic Pituitrin stands without a rival. There is no more active pituitary extract. Pituitrin is standardized by the two accepted methods of determining pituitary activity; the blood-pressure test and the oxytocic test, the latter by use of the isolated uterus. Every lot of Pituitrin represents the same high degree of activity."

Pituitrin is supplied in glaseptic ampoules of 1 c.c. and ½ c.c. capacity, convenient for hypodermatic administration.

---

### ABOUT SOPORIFICS.

The measure of a soporific's usefulness is found not in any single quality but in its adaptability to the various nervous and mental conditions encountered in daily practice. Potency, although it is the first essential, cannot be taken as a standard by which to judge a remedy of this class. Danger either of immediate evil effect or habit-forming too often accompanies this quality. The sphere of usefulness of such remedies is limited to cases which are under the constant supervision of the physician or trained attendant. It is essential to have a safe sedative-hypnotic for the vast majority of cases. Such a remedy must be free from habit-forming drugs and dangerous heart depressants. On the other hand, it must possess sufficient potency to relieve all nervous manifestations and highly excited mental states not absolutely requiring the administration of an opiate. It must be a remedy in which dependence can be placed not only as to positive therapeutic effect, but also as to freedom from deleterious results.

---

**REPRINTS**

Of Original Articles published in the Journal-Record of Medicine will be furnished at the following prices:

**500 Copies 50c per page.**

**1000 Copies 75c per page.**

When Reprints are desired it should be indicated on the copy.

**SAL HEPATICA**

**AN EFFERVESCING**

**SALINE COMBINATION**

**LAXATIVE AND**

**ELIMINANT**

BRISTOL-MYERS CO.

NEW YORK

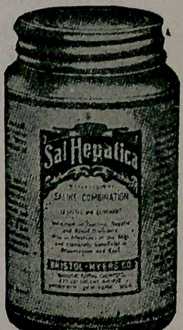

These requirements are fully met in Neurosine. Neurosine is a potent sedative-hypnotic-antispasmodic not dependent on habit-forming drugs or coal tar derivatives for its therapeutic effect. It possesses positive sedative power and this power is made available wherever desired by absolute freedom from danger. This combination of maximum potency and perfect safety with the added advantage of exemption from narcotic laws makes Neurosine the most satisfactory soporific for routine use.

---

### "IN PARTICULAR CASES."

Therapeutic efficiency in the use of the bromides is often as dependent on the avoidance of untoward effects as on the attainment of physiologic activity. For this reason Peacock's Bromides offer the most satisfactory bromide therapy, for not only does this happy combination of carefully selected bromide salts insure all the benefits of the most active bromide preparation, but it does so with the great advantage that gastric disturbance and all tendencies to bromism are reduced to a minimum. This is why in "particular cases" so many physicians are in the habit of insisting on the use of Peacock's Bromides.

---

### CODLIVER OIL FOR PUNY CHILDREN.

Whilst codliver oil long has been recognized as of the utmost value as a nutritive for puny children, yet by reason of the oil's obnoxious taste and odor it had to be dispensed with. It was not until the pharmaceutical chemist made it possible to put codliver oil up in palatable form that its purposes have been utilized to the fullest. Cord. Ext. Ol. Morrhuae Comp. (Hagee) is the standard of the palatable codliver oil products. No part of the oil's worth has been lost in the process of manufacture, and owing to its ease of digestion, it may be continued for long periods. This latter fact makes it of pre-eminent value in the treatment of disability in women and children.

